# Lifting the lid on pilus assembly

**DOI:** 10.7554/eLife.04997

**Published:** 2014-10-28

**Authors:** Han Remaut, Nir Ben-Tal

**Affiliations:** 1 **Han Remaut** is in the VIB Structural Biology Research Center, Brussels, Belgium and is in the Structural Biology Brussels Lab, Vrije Universiteit Brussel, Brussels, Belgiumhan.remaut@vib-vub.be; 2 **Nir Ben-Tal** is in the Department of Biochemistry and Molecular Biology, George S. Wise Faculty of Life Sciences, Tel-Aviv University, Tel-Aviv, Israelbental@tauex.tau.ac.il

**Keywords:** dynamics, outer membrane protein, evolution, structure, *E. coli*

## Abstract

A combination of computer simulations, evolutionary analysis and graph theory has provided new insights into the assembly of pili on the surface of bacteria.

**Related research article** Farabella I, Pham T, Henderson NS, Geibel S, Phan G, Thanassi DG, Delcour AH, Waksman G, Topf M. 2014. Allosteric signalling in the outer membrane translocation domain of PapC usher. *eLife* **3**:e03532. doi: 10.7554/eLife.03532

**Image** An usher protein after activation

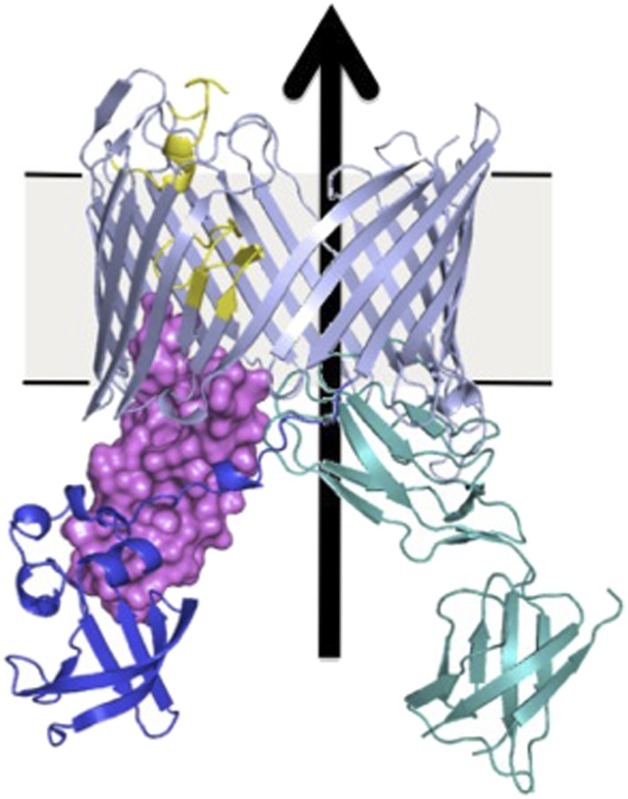

Pathogenic bacteria are coated with adhesive structures that enable them to seek out and colonize their host organisms and tissues of choice. In gram-negative bacteria, these adhesive structures are often long linear protein fibers called pili. Pilus assembly is a marvelous molecular process that requires the orchestrated passage and polymerization of hundreds of subunits across the two lipid bilayers that make up the cell envelope.

In principle, the ability to polymerize and form fibers of a specific ordering is intrinsic to the pilus subunits (Vetsch et al., 2004; Rose et al., 2008). In bacterial cells, however, two proteins facilitate and catalyze this process: one is a chaperone protein that resides in the periplasm (the space between the two lipid bilayers); the other is an usher protein that is embedded in the outer membrane. The role of the chaperone protein is to maintain pilus subunits in a higher energy conformation that is suitable for polymerization (Sauer et al., 1999, 2002).The role of the usher protein is to catalyze the polymerization and to form a barrel-shaped channel for the passage of the growing pilus to the cell surface (Nishiyama et al., 2008; Phan et al., 2011).

An unresolved question is how the assembly process at the usher protein is switched on. Both in vitro and in cells, the usher protein is a highly efficient catalyst, but only after it has been activated—that is, only after a plug domain that blocks the usher channel has been moved out of the way (Figure 1). This plug is important because, without it, the usher channel would form a 4 nm puncture in the outer membrane that would compromise its insulating function (Remaut et al., 2008). When the usher protein has been activated, the growing pilus takes its place in the usher channel.

**Figure 1.**
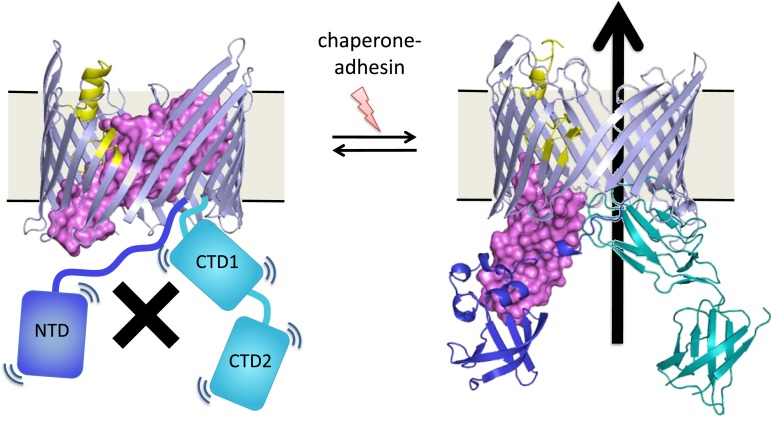
Many bacteria are covered with long protein fibers called pili that contain hundreds of pilus subunits. These pili are assembled at usher proteins (above) that are embedded in the cell walls of bacteria; chaperone proteins are also involved in the assembly process. The usher protein recruits chaperone-subunit complexes, catalyzes the polymerization of the subunits, and allows passage of the pili to the cell surface. The usher protein must be activated in order to act as a catalyst; activation involves moving a ‘plug’ (purple) that blocks the channel in the protein (left) through an angle of ∼150° to open the channel (right). The movement of the plug also orients the domains (NTD, CTD1 and CTD2) that recruit the chaperone-subunit complexes (not shown). These models are based on X-ray structures of the PapC usher protein (PDB:2vqi; Remaut et al., 2008) and the FimD usher protein bound to a chaperone-subunit complex (PDB:3rfz; Phan et al., 2011; Geibel et al., 2013). Farabella et al. explored the roles of the alpha-helix (yellow) and the beta-hairpin (blue/grey) in the activation process.

Triggering activation of the usher protein involves the binding of the first chaperone-subunit complex. X-ray structures of an activated FimD usher protein engaged with growing pilus intermediates showed that the plug domain had swung into the periplasm to vacate the usher channel and to help position the domains that recruit the chaperone-subunit complexes (Figure 1; Phan et al., 2011; Geibel et al., 2013). However, the details of the processes that control how the plug swings through an angle of ∼150° are not fully understood.

Now, in *eLife*, Maya Topf and co-workers—including Irene Farabella as first author, and co-workers at Birkbeck College, University College London, the University of Houston and Stony Brook University—use a unique computational approach to model the pathway that leads to usher activation (Farabella et al., 2014). This approach involves detecting the key amino acids that are involved in controlling the position of the plug in the channel.

To allow the plug to move, the binding interactions of the plug in the channel need to be only marginally stable: such situations are difficult to compute. Furthermore, this metastability is the result of a large number of weak interactions, rather than being the result of a few strong interactions, which is typical for proteins (Kessel and Ben-Tal, 2011). Topf and co-workers designed unique computational approach to circumvent these modeling difficulties. Previous studies suggested that this process is controlled by two regions: an alpha-helix and a beta-hairpin (Figure 1). Therefore, Farabella et al. started by performing molecular dynamics simulations of the native protein and of mutant proteins in which the alpha-helix and/or the beta-hairpin are deleted. They recorded the polar interactions (that is, hydrogen bonds and salt bridges) that were frequently observed in the simulations, and compared the frequencies of these interactions in the native protein and in the mutants.

Farabella et al. used evolutionary data to highlight particularly important interactions, assuming that the interacting amino acids should be conserved (Ashkenazy et al., 2010). Evolutionary data were also used to detect couplings (co-evolution) where both amino acids change in tandem to retain the interaction (Marks et al., 2012; de Juan et al., 2013). To give an example, the importance of a salt bridge could be detected if the two oppositely charged amino acids involved in the interaction, say arginine and glutamate, are strictly conserved. However, even if the interacting amino acids evolve, the interaction would persist if every arginine-to-glutamate substitution was balanced by a glutamate-to-arginine substitution. Thus, both evolutionary conservation and coupling could be indicative of importance.

The results were presented as a network with edges between amino acids that interact with each other. The network was inhomogeneous with some regions containing more interactions than others. Further analysis, using techniques based on graph theory, showed that the network contained ‘communities’ of amino acids: five of these communities were particularly large and also included interactions between amino acids that were separated by relatively large distances. Farabella et al. then performed experiments to check if these communities were involved in “gating” the channel in the usher protein. Amino acid residues from four of the five communities were involved. Moreover, some of these amino acids were from regions of the protein that, based on the X-ray structures alone, did not stand out as important. Also of note, amino acids close to each other in a community can have opposing effects on gating, resulting either in the stabilization of closed pores, or in pores remaining open for longer, which makes the bacteria more sensitive to antibiotics.

The activation and inactivation of many more transmembrane channels and signaling complexes is based on switching between conformations of very similar energy. The similarity is required by design in order to allow a response to relatively small external signals; if one conformation were significantly more stable than the other, the system would get stuck. The methodology developed by Farabella et al. provides a powerful tool for the identification of the intramolecular components that control these equilibria. For pilus assembly, the incoming chaperone-subunit complex also contains intermolecular cues that help to trigger the activation of the usher protein (Munera et al., 2008). It is not clear if these intramolecular and intermolecular elements interact with each other: answering this question will require further structural insights, ideally of an usher-chaperone-subunit complex before activation.
